# A network pharmacology approach confirms Biejiaxiaozheng pills combat hepatic fibrosis by modulating macrophage inflammation and hepatic stellate cell activation

**DOI:** 10.1038/s41598-025-09002-1

**Published:** 2025-07-09

**Authors:** Weibin Wu, Yanli Liao, Jiefei Liang, Mi Huang, Guifeng Zhang, Yuying Hu, Chao Yuan, Duanzhuo Li

**Affiliations:** 1 School of Basic Medicine, Zhaoqing Medical College, Zhaoqing, Guangdong China; 2 School of Public Health, Zhaoqing Medical College, Zhaoqing, Guangdong China; 3 School of Pharmacy, Zhaoqing Medical College, Zhaoqing, Guangdong China; 4 School of Traditional Chinese Medicine, Zhaoqing Medical College, Zhaoqing, Guangdong China; 5 Department of Scientific Research and Experiment Center, Zhaoqing Medical College, Zhaoqing, Guangdong China

**Keywords:** Biejiaxiaozheng pills, Network pharmacology, Macrophage, Inflammation, HSCs, Fibrosis, Pharmacology, Target validation, Liver fibrosis

## Abstract

Biejiaxiaozheng (BJXZ) pills, a traditional Chinese medicine, have demonstrated anti-fibrotic effects; however, their mechanisms in treating liver fibrosis remain unclear. This study aimed to explore the active targets and underlying mechanisms of BJXZ pills using network pharmacology and experimental validation. Network pharmacology analysis identified 213 common drug-disease targets, 1131 Gene Ontology terms, and 163 signaling pathways (including the TNF pathway). Experimental results showed that BJXZ pills significantly suppressed LPS-induced viability increase in RAW264.7 macrophages by inhibiting NF-κB activation (p-P65), reducing INOS expression, and decreasing inflammatory cytokines. They also alleviated oxidative stress by upregulating Nrf2 and HO-1, restoring mitochondrial membrane potential, and enhancing ATP production. Additionally, BJXZ pills markedly inhibited TGF-β-induced activation of LX-2 hepatic stellate cells, reducing fibrotic markers like α-SMA. These findings suggest that BJXZ pills exert anti-fibrotic effects via multiple pathways, with the TNF pathway playing a key role. Mechanistically, the protective effects involve suppressing macrophage and hepatic stellate cell activation, highlighting BJXZ pills as a promising therapeutic option for liver fibrosis.

**Supplementary Information:**

The online version contains supplementary material available at https://doi.org/10.1038/s41598-025-09002-1.

## Introduction

Liver fibrosis is a liver disease induced by chronic hepatocyte damage due to non-alcoholic fatty liver disease and hepatitis B. The primary presentation of liver fibrosis is excessive deposition of hepatocyte extracellular matrix. Hepatic stellate cells are the primary effector cells responsible for the development of liver fibrosis. Cytokines secreted by Kupffer cells in the liver activate hepatic stellate cells, which stimulates their increased proliferation and synthesis of extracellular matrix synthesis, as well as decreased extracellular matrix degradation, thereby gradually causing fibrosis ^1,2^.

Biejiaxiaozheng (BJXZ) pills is a traditional Chinese medicine pill composed of *Trionyx sinensis* Wiegmann, musk, *Curcua kwangsiensis* S.G.Lee et C.FLiang, *Sparganium stoloniferum* Buch.-Ham, *Panax quiquefolium* L., *Astragalus membranaceus* (Fisch.) Bge.var. mongholicus (Bge.) Hsiao, *Atractylodes macrocephala* Koidz., *Poria cocos* (Schw.) Wolf, *Paeonia lactiflora* Pall., *Rheum palmatum* L., and *Glycyrrhiza uralensis* Fisch. Basic research has shown that the aforementioned components have anti-fibrotic effects ^3–6^. This drug has been used in clinical practice in China for more than 30 years and has a suitable efficacy in treating liver fibrosis. Our previous research demonstrates that BJXZ pills was capable of reversing rat liver fibrosis induced by carbon tetrachloride and AML12 mouse hepatocyte injury induced by TGF-β through alleviating inflammatory responses and enhancing antioxidant stress mechanisms ^7,8^.

Network pharmacology is based on the principle of traditional Chinese medicine active ingredient mining and target prediction and employs traditional Chinese medicine chemistry, genomic, and target databases to study the active ingredients in traditional Chinese medicine. Identification of the active ingredients in a particular medicinal compound allows for the prediction of potential targets and the construction of a drug–target–disease network. For this network, the underlying pharmacological mechanisms, including the components, targets, and pathways, can be predicted ^9–11^.

To understand the possible mechanisms and pathways underlying the reversal of liver fibrosis by Biejiaxiaozheng pills, we employed network pharmacology to analyze and mine the potential targets of Biejiaxiaozheng pills. We then utilized an in vitro cell model to validate the effects of the pathways obtained in the network pharmacology analysis results and demonstrate the reliability and accuracy of these results.

## Materials and methods

### Cell line and culture

The RAW 264.7 mouse monocyte/macrophage cell line and the LX-2 human hepatic stellate cell lines were purchased from the Chinese Academy of Sciences Cell Bank. The culture protocol recommended by the Chinese Academy of Sciences Cell Bank was used as a reference for the culture of RAW 264.7 and LX-2 cell lines. Fetal bovine serum (10% or 2%) was added to high-glucose DMEM medium to prepare the complete culture medium. The cells were cultured in an incubator at 37 °C with 5% CO2, and cells in the logarithmic growth phase were used for the study.

### Reagents

The following reagents were used in this study: Biejiaxiaozheng pills was obtained from Zhaoqing Traditional Chinese Medicine Hospital; Ethanol anhydrous(#E809056), Tris Buffered Saline with Tween 20 (#T854550) were purchased from Macklin(Shanghai, China); High glucose DMEM complete medium (#PM150210B) was purchased from Procell(Wuhan, China); 0.45 μM PVDF membrane(#IPVH00010) and 0.22 μM micropore filter(#SLGP033R) were purchased from Merck (Darmstadt, Germany); TNF-α(#1217202), IL-1β(#1210122), IL-6(#1210602), and MCP-1(#1217392) ELISA kits were purchased from Dakewe (Guangdong, China); Human transforming growth factor-β (TGF-β, #CA59) was purchased from Novoprotein (Suzhou, China); 96-Well White Opaque Plates (#FCP968), CCK-8 assay kits(#C0039), luminescent cell viability assay kit(#C0065), Reactive Oxygen Species Assay Kit(#S0033), Mitochondrial Membrane Potential Assay Kit with Rhodamine 123(#C2008), Cell lysis buffer(#P0013), Nuclear and Cytoplasmic Protein Extraction Kit(#P0027), protease and phosphatase inhibitor cocktail(#P1045), SDS-PAGE Sample Loading Buffer(#P0015), Prestained Color Protein Marker(#P0075), Soaking and Activation Buffer for PVDF Membrane (#P0021S), BSA (#ST023), Blocking Buffer(#P0023B), ECL kit(#P0018) were purchased from Beyotime(Shanghai, China); Nrf2(#AF0639), HO-1(#AF5393), P65(#AF5006), p-P65(#AF2006), INOS(#AF0199), DRP1(#DF7037), OPA1(#DF8587), Smad 2(#AF6449), p-Smad 2(#AF3449), Collagen type I(#AF7001), Collagen type III(#AF5457), α-smooth muscle actin (α-SMA)(#AF1032), Vimentin (#AF7013), Fibronectin(#AF5335), β-Tubulin(#AF7011), Histone H3(#AF0863) antibodies, and Goat Anti-Rabbit IgG (H+L) HRP(#S0001) were purchased from Affinity (Jiangsu, China).

### Collection of Biejiaxiaozheng pill active chemicals and target prediction

The TCMSP database (https://old.tcmsp-e.com/tcmsp.php) was used to search for targets of nine plant components in Biejiaxiaozheng pills. The drug screening criteria were oral bioavailability (OB) ≥ 30% and drug-likeness (DL) ≥ 0.18. The targets constituents of the plant components in Biejiaxiaozheng pills were obtained. Finally, the STRING database was used to convert the target proteins of traditional Chinese medicine components into gene IDs. The Materia Medica HERB website (http://herb.ac.cn/) was used to collect the active ingredients of *Trionyx sinensis* Wiegmann and musk in Biejiaxiaozheng pills by using the keywords BIE JIA and SHE XIANG, respectively. The related genes were obtained from the STRING Database.

### Liver fibrosis disease target prediction

The keyword “liver fibrosis” was used to search for effector targets of liver fibrosis in the GeneCards database (https://www.genecards.org/) and DisGeNET database (https://www.disgenet.org/). The corresponding genes of drug targets and liver fibrosis were inputted into the BioinfoGP website, and Venny 2.1 (https://bioinfogp.cnb.Csic.es/tools/venny/index.html) was used to obtain the common target genes of liver fibrosis influenced by Biejiaxiaozheng pills.

### Construction of protein interaction network

The common target genes were inputted into the STRING database (https://string-db.org/). Multiple proteins was selected, and species were restricted to Homo sapiens in order to obtain the common target PPI network data. The TSV file was downloaded and inputted into the Cytoscape 3.9.0 software and combined with the CentiScaPe and Analyze Network plugins for data analysis and plotting of the PPI network.

### GO biological function analysis and KEGG pathway enrichment analysis

The DAVID database was used for GO functional analysis and KEGG pathway enrichment analysis of key targets in order to further understand the functions of potential target genes affected by Biejiaxiaozheng pills during liver fibrosis treatment, in addition to their roles in signaling pathways. The common targets of Biejiaxiaozheng pill treatment for liver fibrosis were inputted into the DAVID database. “OFFICIAL_GENE_SYMBOL” was selected for a Select Identifier, “Homo sapiens” was selected for Select species, and “Gene List” was selected for List Type. At the same time, a statistically significant difference with *P* < 0.05 was set as the screening criterion for GO analysis and KEGG analysis ^12–14^. Data was inputted into the bioinformatics online tool (http://www.bioinformatics.com.cn/) to plot the top items in the enrichment analysis by gene number into a histogram.

### Component-disease-target-pathway network construction and analysis

Excel was used to sort the Chinese medicinal materials in Biejiaxiaozheng pills, as well as the active ingredients, corresponding targets, and corresponding effector targets in the key pathways. After that, the data was inputted into the Cytoscape 3.9.0 software to construct a drug–component-disease-target-pathway network map. The Network Analyzer and CentiScaPe plugins were used for topological analysis.

### Drug preparation

Biejiaxiaozheng pills dry powder (50 g) was dissolved in 100 mL of ultra-pure water and sonicated for extraction. The extraction was repeated three times, and then the filtrates were combined. The combined solution underwent rotary evaporation to obtain a concentrated suspension. The suspension was then lyophilized to obtain a uniform drug powder. Then 100 mg of lyophilized powder was dissolved in 10 mL of basal culture medium to prepare a 10 g·L^−1^ stock solution, and then based through a 0.22 μm filter membrane for sterilization.

### CCK-8 assay measurement of the effects of BJXZ pills on the viability of RAW 264.7 and LX-2 cells

Study on the toxicity of the drug on RAW 264.7 and LX-2 cells. RAW 264.7 and LX-2 cells in the logarithmic growth phase were seeded into a 96-well plate. After that, various concentrations (0–5 g·L^−1^ for RAWA 264.7 and 0–1 g·L^−1^ for LX-2) of Biejiaxiaozheng pill solution were added to the wells. Following treatment (24 h for RAW 264.7 and 48 h for LX-2), the culture medium was changed to complete culture medium containing 10% CCK-8 and incubated for 15 min. After that, a multi-mode microplate reader (SPARK, Tecan) was used to measure the absorbance of every well at 450 nm as the detection wavelength and 620 nm as the reference wavelength. Finally, Asample = A_450_–A_620_ was used as the final absorbance value.

Effects of drugs on lipopolysaccharide (LPS)-elevated RAW 264.7 cell activity. Cells were seeded in a 96-well plate and divided in five groups: (1) control group, (2) model group (LPS 1 mg·L^−1^), (3) Biejiaxiaozheng pill 0.2 g·L^−1^ group (LPS 1 mg·L^−1^ + Biejiaxiaozheng pill 0.2 g·L^−1^), (4) Biejiaxiaozheng pill 0.5 g·L^−1^ group (LPS 1 mg·L^−1^ + Biejiaxiaozheng pill 0.5 g·L^−1^) , and (5) Biejiaxiaozheng pill 1 g·L^−1^ group (LPS 1 mg·L^−1^ + Biejiaxiaozheng pill 1.0 g·L^−1^). The various drug concentrations were added to the corresponding wells. After 24 h of treatment, the absorbance was measured from the plate using the same method as described above for detection.

Effects of drugs on TGF-β-induced LX-2 cell. Cells were seeded in a 96-well plate and divided in five groups: (1) control group, (2) model group (TGF-β 10 μg·L^−1^), (3) Biejiaxiaozheng pill 0.1 g·L^−1^ group (TGF-β 10 μg·L^−1^ + Biejiaxiaozheng pill 0.1 g·L^−1^), (4) Biejiaxiaozheng pill 0.2 g·L^−1^ group (TGF-β 10 μg·L^−1^ + Biejiaxiaozheng pill 0.2 g·L^−1^) , and (5) Biejiaxiaozheng pill 0.5 g·L^−1^ group (TGF-β 10 μg·L^−1^ + Biejiaxiaozheng pill 0.5 g·L^−1^). The various drug concentrations were added to the corresponding wells. After 48 h of treatment, the absorbance was measured from the plate using the same method as described above for detection.

### ELISA measurement of the effects of Biejiaxiaozheng pills on the expression of TNF-α, IL-1β, IL-6, and MCP-1 in RAW 264.7 cell culture medium

RAW 264.7 cells in the logarithmic growth phase were seeded in 12-well plates and grouped according to LPS-elevated model. The various drug concentrations were added to the corresponding wells. After 24 h of treatment, the cell culture supernatant was collected, and the levels of TNF-α, IL-1β, IL-6, and MCP-1 in the supernatant were measured according to the manufacturers’ instructions of the kits.

### DCFA-DA assay measurement of the effects of Biejiaxiaozheng pills on the level of intracellular reactive oxygen species (ROS) in RAW 264.7 cells

RAW 264.7 cells in the logarithmic growth phase were seeded in 6-well plates and grouped according to LPS-elevated model. The various concentrations of the drug were added to the corresponding wells. After 6 h of treatment, the supernatant was discarded, and the basal culture medium containing DCFH-DA was added. The plates were incubated for 30 min before discarding the supernatant, and PBS was used to gently wash the wells three times. Then 1 mL of basal culture medium was added to every well, and the cells were collected and analyzed with a flow cytometer (CytoFLEX, Beckman Coulter).

### Rhodamine 123 measurement of the effects of Biejiaxiaozheng pills on the mitochondrial membrane potential of RAW 264.7 cells

RAW 264.7 cells in the logarithmic growth phase were seeded in 6-well plates and grouped according to the apoptosis model. The various drug concentrations were added to the corresponding wells. After 24 h of treatment, the supernatant was discarded, and the basal culture medium containing Rhodamine 123 was added. The plates were incubated for 15 min before discarding the supernatant, and PBS was used to gently wash the wells three times. Then 1 mL of basal culture medium was added to every well, and the cells were collected and analyzed with the flow cytometer.

### Chemiluminescence measurement of the effects of Biejiaxiaozheng pills on the intracellular ATP content in RAW 264.7 cells

RAW 264.7 cells in the logarithmic growth phase were seeded into a white 96-well plate. The cells were grouped as LPS-elevated model. The various drug concentrations were added to the corresponding wells, and the cells were treated for 24 h. The test solution was added according to the manufacturer’s instructions, and the plate was incubated in the incubator for 10 min. After that, a multi-mode microplate reader was used to measure the chemiluminescence intensity of each well. The integration time was set to 10 ms.

### Western blot measurement of the effects of Biejiaxiaozheng pills on protein expression in RAW 264.7 and LX-2 cells

Pre-cooled PBS was used to collect cells, and protein samples were made. After that, the samples were loaded onto an 8% polyacrylamide gel and electrophoresis was carried out, followed by membrane transfer at 200 mV for 90 min. The membranes were blocked using blocking solution at 25 °C for 30 min, and then they were incubated with primary antibodies (1:1000) in a shaking incubator at 4 °C for 12h. Then TBST was used to wash the membranes four times, and 4% BSA solution was used to prepare the secondary antibody (1:5000). The membranes were incubated at room temperature for 60 min. TBST was used to wash the membranes four times prior to analyzing the membranes via a western blot imaging system (iBright FL1000, Invitrogen).

### Statistical analysis

ImageJ (https://imagej.nih.gov/ij/) was used for semi-quantitation of image data. Quantitative data was expressed as the mean ± standard deviation ($\overline{x }$± s). SPSS 25.0.0 was used for data analysis, GraphPad Prism 8 was used for graph plotting, One-way ANOVA was used for multiple group testing, and variance homogeneity test was carried out. A difference of *P* < 0.05 was considered to be statistically significant and are marked with an asterisk (*) in the figure.

## Results

### Network pharmacology predicts the targets of Biejiaxiaozheng (BJXZ) pills on liver fibrosis

**Fig. 1:**
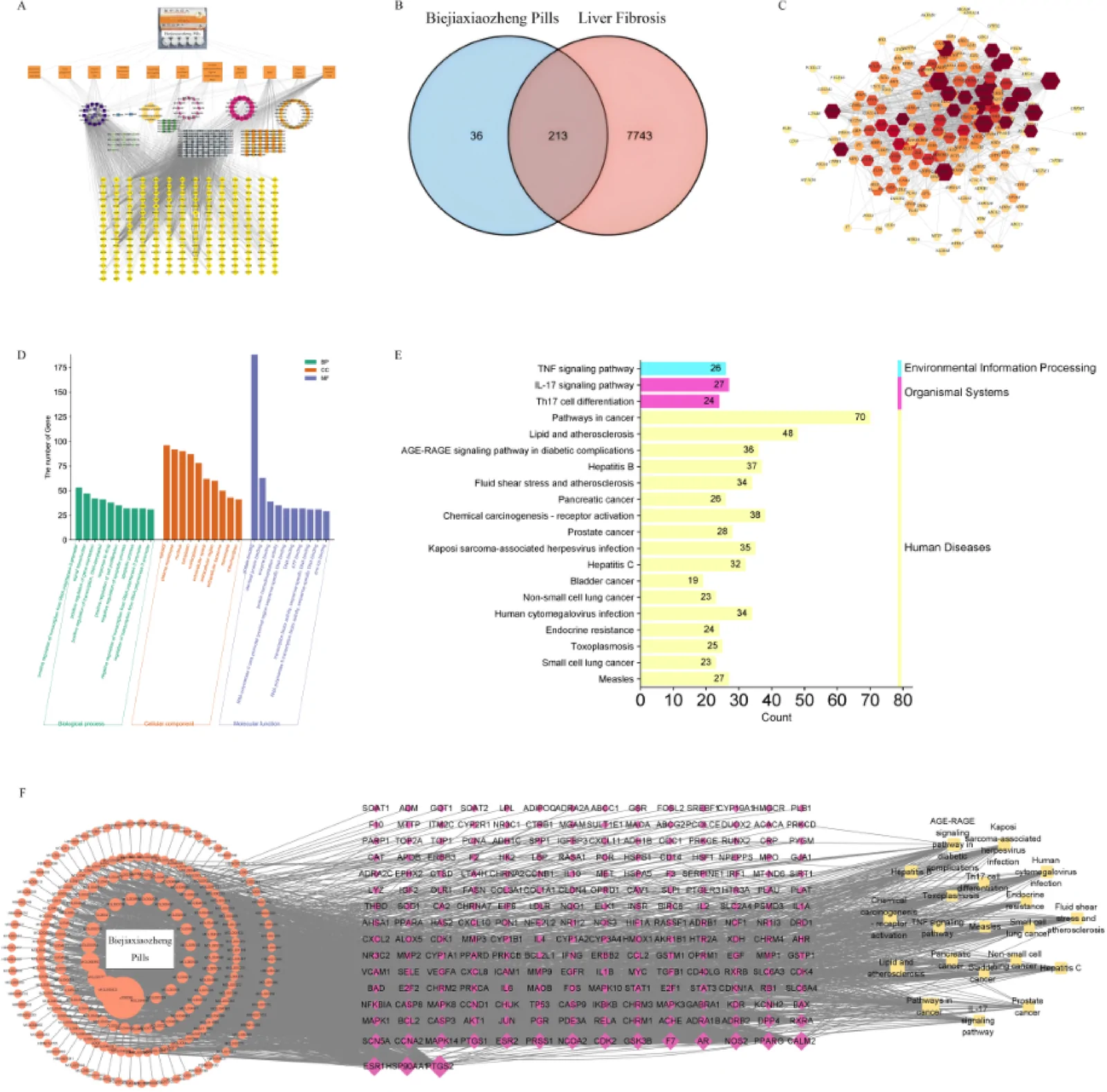
Network pharmacology analysis of underlying mechanisms of Biejiaxiaozheng pills in liver cirrhosis. (**A**) The ingredients and target network of BiejiaXiaozheng pills. (**B**) Prediction of therapeutic targets of BiejiaXiaozheng pills for liver fibrosis. (**C**) The PPI network of therapeutic targets of BiejiaXiaozheng pills for liver fibrosis. (**D**) GO analysis according to BiejiaXiaozheng pills for liver fibrosis. (**E**) KEGG analysis revealed the pathways related to BiejiaXiaozheng pills for liver fibrosis. (**F**) Component–disease–target–pathway network of BiejiaXiaozheng pills for liver fibrosis.

The study identified 250 active ingredients in BJXZ pills through the TCMSP and HERB databases (Supplementary Table 1 and 2), targeting 249 unique genes, with network analysis revealing interactions between these components and liver fibrosis-related genes. The active ingredients of BJXZ pills and corresponding targets were constructed a traditional Chinese medicine-component-target network. In the Fig. 1A, the yellow nodes represent targets, the orange squares represent the active ingredients of BJXZ pills, and the other nodes represent traditional Chinese medicine active ingredients. The retrieval of liver fibrosis-related genes from the GeneCards and DisGeNET databases yielded 7956 genes. Subsequently, these were cross-referenced with the 249 drug-target genes identified in Fig. 1A, resulting in 213 overlapping target genes (Fig. 1B). Protein–protein interaction (PPI) analysis of 213 overlapping targets demonstrated significant enrichment (*P* < 1.0^e−16^), highlighting 42 core targets, including TP53, STAT3, JUN, AKT1, MAPK3, IL6, CASP3, EGFR, RELA and MAPK1 (Fig. 1C). GO enrichment analysis of the 213 overlapping targets identified 1,131 GO terms, including 848 associated with biological processes (BP), 184 with molecular functions (MF), and 99 with cellular components (CC). The top 10 most significantly enriched terms were selected for visualization (Fig. 1D). KEGG pathway enrichment analysis identified 176 pathways, with the top 20 including the TNF signaling pathway, IL-17 signaling pathway, Th17 cell differentiation, and disease-associated pathways such as lipid and atherosclerosis, AGE-RAGE signaling in diabetic complications, and pathways in cancer (Fig. 1E). A traditional Chinese medicine component–disease–target–pathway network (435 nodes, 3021 edges) further emphasized the role of inflammatory (TNF, IL-17), metabolic (lipid metabolism), and oncogenic (cancer pathways, endocrine resistance) signaling pathways in mediating the pharmacological effects of BJXZ pills on liver fibrosis (Fig. 1F).

### Effect of BJXZ pills on LPS-induced inflammatory model in RAW 264.7 cells

**Fig. 2:**
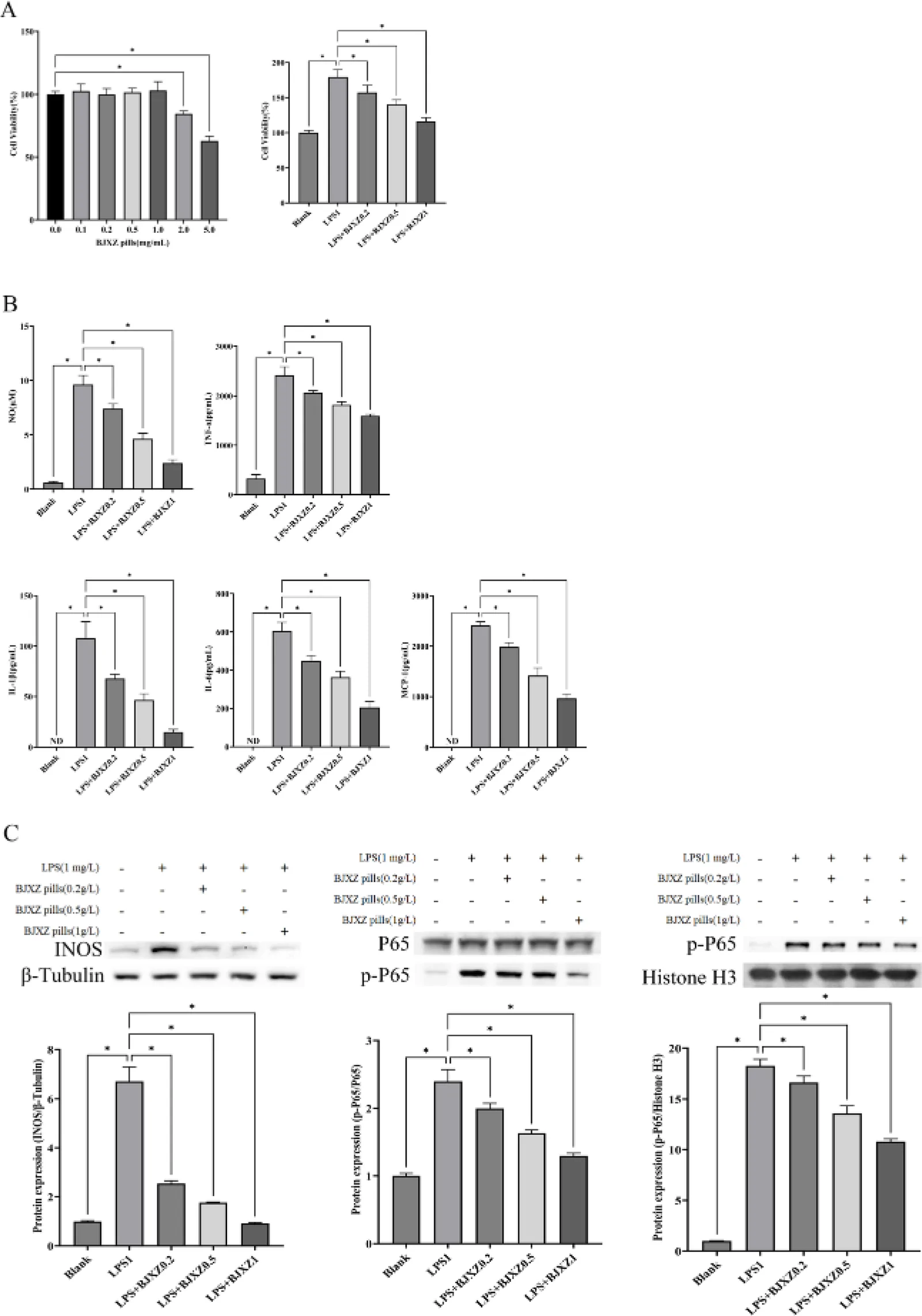
Effect of Biejiaxiaozheng pills on the LPS-induced inflammatory model of RAW 264.7 cells. (**A**) Effect of Biejiaxiaozheng pills on the Viability of RAW 264.7 Cells. (**B**) Effect of Biejiaxiaozheng pills on the contents of inflammatory factors in the Supernatant of RAW 264.7 Cells. (**C**) Effect of Biejiaxiaozheng pills on the Phosphorylation and nuclear translocation of p65 and INOS Protein Expression of RAW 264.7 Cells.

Based on the aforementioned results of network pharmacology analysis and previous research ^7^, we speculate that the TNF pathway is one of the crucial pathways for the anti-hepatic fibrosis effect of BJXZ pills. Hence, we used LPS to induce RAW 264.7 cells to establish an inflammation model, to verify the effect of BJXZ pills on the TNF signaling pathway. We initially employed the CCK-8 method to examine the toxicity of BJXZ pills on RAW 264.7 cells, with the aim of determining the drug dosage range for subsequent research. The findings indicated that BJXZ pills at a concentration of 2 g·L^−1^ or above could markedly reduce the viability of RAW 264.7 cells. Consequently, the drug dosage range for the subsequent research will be chosen at concentrations of 1 g·L^−1^ and below. After the drug dosage was determined, we utilized LPS to induce RAW 264.7 cells to establish an inflammation model. Firstly, the CCK-8 method was employed to examine the influence of BJXZ pills on the cell viability of the inflammation model. The results demonstrated that, in comparison with the blank group, after induction with 1 mg·L^−1^ lipopolysaccharide in the LPS group, the viability of RAW 264.7 cells was significantly elevated (*P* < 0.05). In contrast to LPS, BJXZ pills could significantly reduce the viability of RAW 264.7 cells in a dose-dependent manner, suggesting that BJXZ pills have an inhibitory effect on the M1 polarization of macrophages induced by LPS (Fig. 2A).

Subsequently, we employed ELISA to determine the contents of inflammatory factors in the culture supernatant of the RAW264.7 cell inflammation model and Western blotting to detect the phosphorylation level of the p65 protein, to assess the impact of BJXZ pills on the NF-κB pathway function of the RAW264.7 cells inflammation model. The results demonstrated that, in contrast to the blank group, the inflammatory factors such as NO, TNF-α, IL-1β, IL-6, and MCP-1 in the culture supernatant of the LPS group were significantly elevated (*P* < 0.05), while BJXZ pills could dose-dependently and significantly decrease (*P* < 0.05) the contents of the aforementioned inflammatory factors in the culture supernatant (Fig. 2B).

In the Western blot analysis, it was observed that, compared to the blank group, both the phosphorylation level and nuclear translocation of p65 protein and the expression level of INOS protein were significantly elevated in the LPS group (*P* < 0.05). Conversely, BJXZ pills demonstrated a dose-dependent reduction in both p65 phosphorylation, nuclear translocation and INOS expression levels (*P* < 0.05). These findings suggest that BJXZ pills may inhibit macrophage-mediated inflammatory responses induced by LPS through suppression of NF-κB pathway activation and downregulation of INOS expression (Fig. 2C).

### Effect of BJXZ pills on ROS Levels in LPS-induced inflammation model of RAW 264.7 Cells

**Fig. 3:**
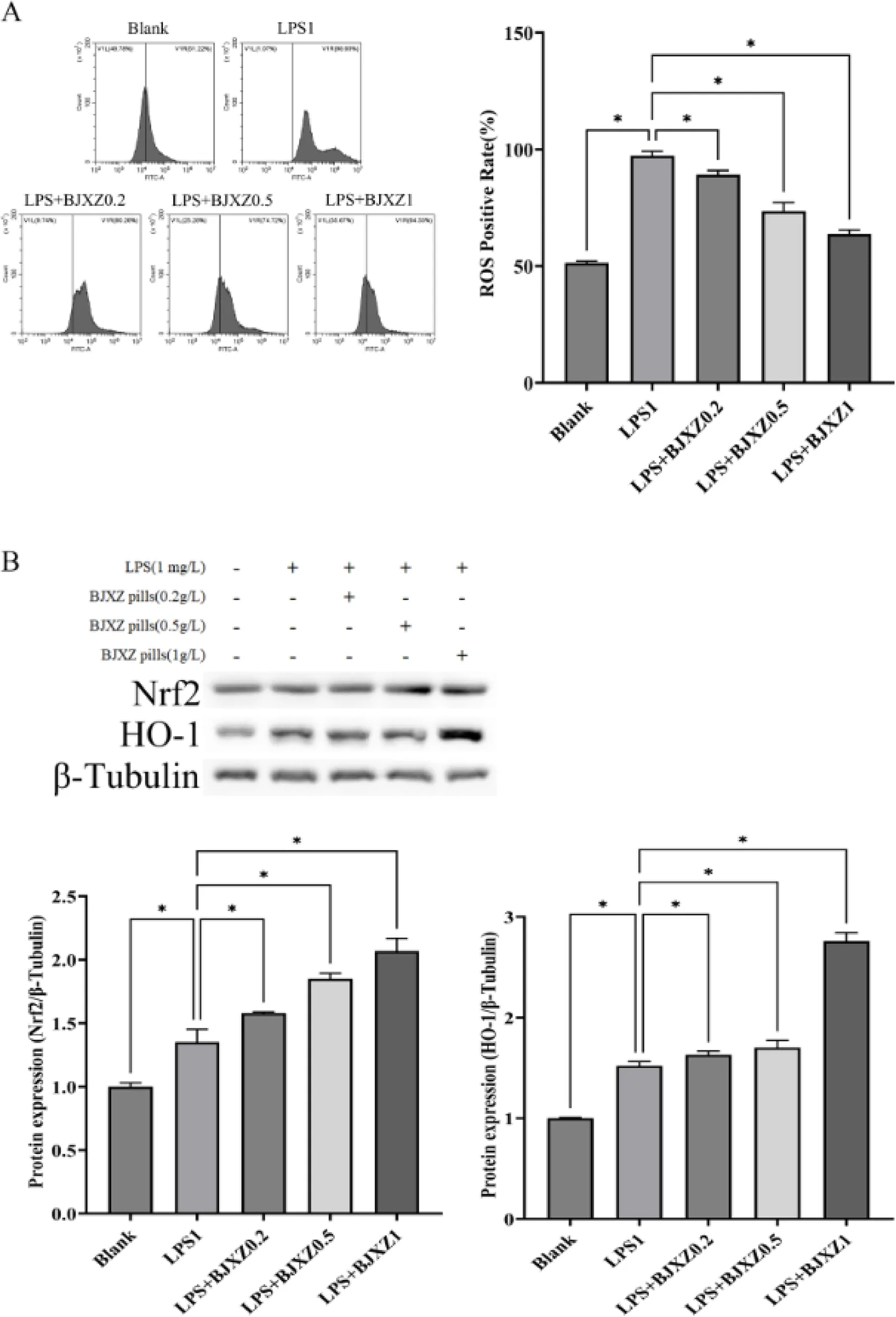
Effect of Biejiaxiaozheng pills on intracellular oxidative stress of RAW 264.7 cells. (**A**) Effect of Biejiaxiaozheng pills on intracellular ROS content of RAW 264.7 Cells. (**B**) Effect of Biejiaxiaozheng pills on the expression of Nrf2 and HO-1 proteins of RAW264.7 cells.

To further explore the anti-inflammatory mechanism of BJXZ pills, we utilized the DCFH-DA and Western blotting to conduct research on the effects of BJXZ pills on the oxidative stress pathway in the RAW 264.7 cell inflammation model. The results demonstrated that, compared with the blank group, the intracellular ROS levels were significantly increased (*P* < 0.05) in the LPS group. BJXZ pills were able to significantly decrease (*P* < 0.05) the intracellular ROS levels in a dose-dependent manner (Fig. 3A). The mechanism might be associated with the fact that BJXZ pills significantly upregulates the expression of Nrf2 and HO-1 protein (*P* < 0.05), and ultimately achieving the enhancement of the cellular antioxidant stress function (Fig. 3B).

### Effect of BJXZ pills on Mitochondrial Function in LPS-induced inflammation model of RAW 264.7 cells

**Fig. 4:**
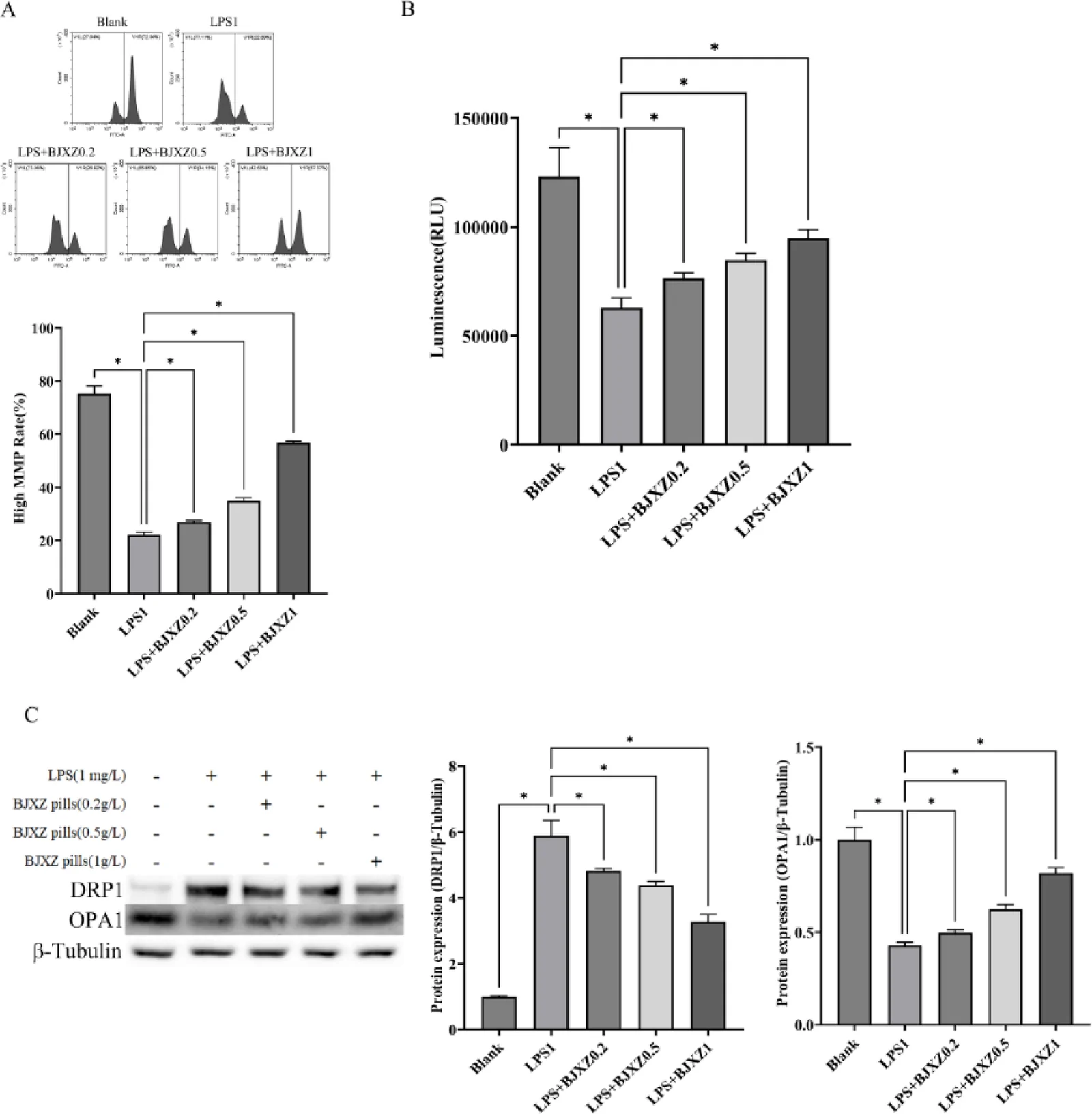
Effect of Biejiaxiaozheng pills on the Mitochondrial Function of RAW264.7 cells. (**A**) Effect of Biejiaxiaozheng pills on the mitochondrial membrane potential of RAW 264.7 cells. (**B**) Effect of Biejiaxiaozheng pills on the ATP contents of RAW 264.7 Cells. (**C**) Effect of Biejiaxiaozheng pills on the expression of DRP1 and OPA1 of RAW264.7 cells.

During the process of the inflammatory response in macrophages, their metabolic pathways transform into glycolysis, namely the Warburg effect. This transformation is beneficial for macrophages to meet the requirements of their activated state and inflammatory response, but it also impairs mitochondrial function and ultimately has an impact on the energy supply of macrophages. We employed Rhodamine 123, chemiluminescence assays, and Western blotting to assess mitochondrial membrane potential, intracellular ATP levels, and the expression of DRP1 and OPA1 proteins in a RAW 264.7 cell inflammation model. This approach aimed to evaluate the effects of BJXZ pills on macrophage energy metabolism under inflammatory conditions. The results indicated that compared to the blank group, both mitochondrial membrane potential (Fig. 4A) and intracellular ATP content (Fig. 4B) were significantly reduced in the LPS group (*P* < 0.05). In contrast, BJXZ pills effectively restored mitochondrial membrane potential and ATP levels in a dose-dependent manner. Furthermore, Western blot analysis revealed that DRP1 protein expression was significantly upregulated while OPA1 protein expression was notably downregulated in the LPS group compared to the blank group; however, BJXZ pills could dose-dependently inhibit DRP1 expression while restoring OPA1 levels. These findings suggest that BJXZ pills enhance energy supply in macrophages during inflammatory states by modulating mitochondrial function (Fig. 4C).

### Effect of BJXZ pills on TGF-β-induced activation model of LX-2 Cells

**Fig. 5:**
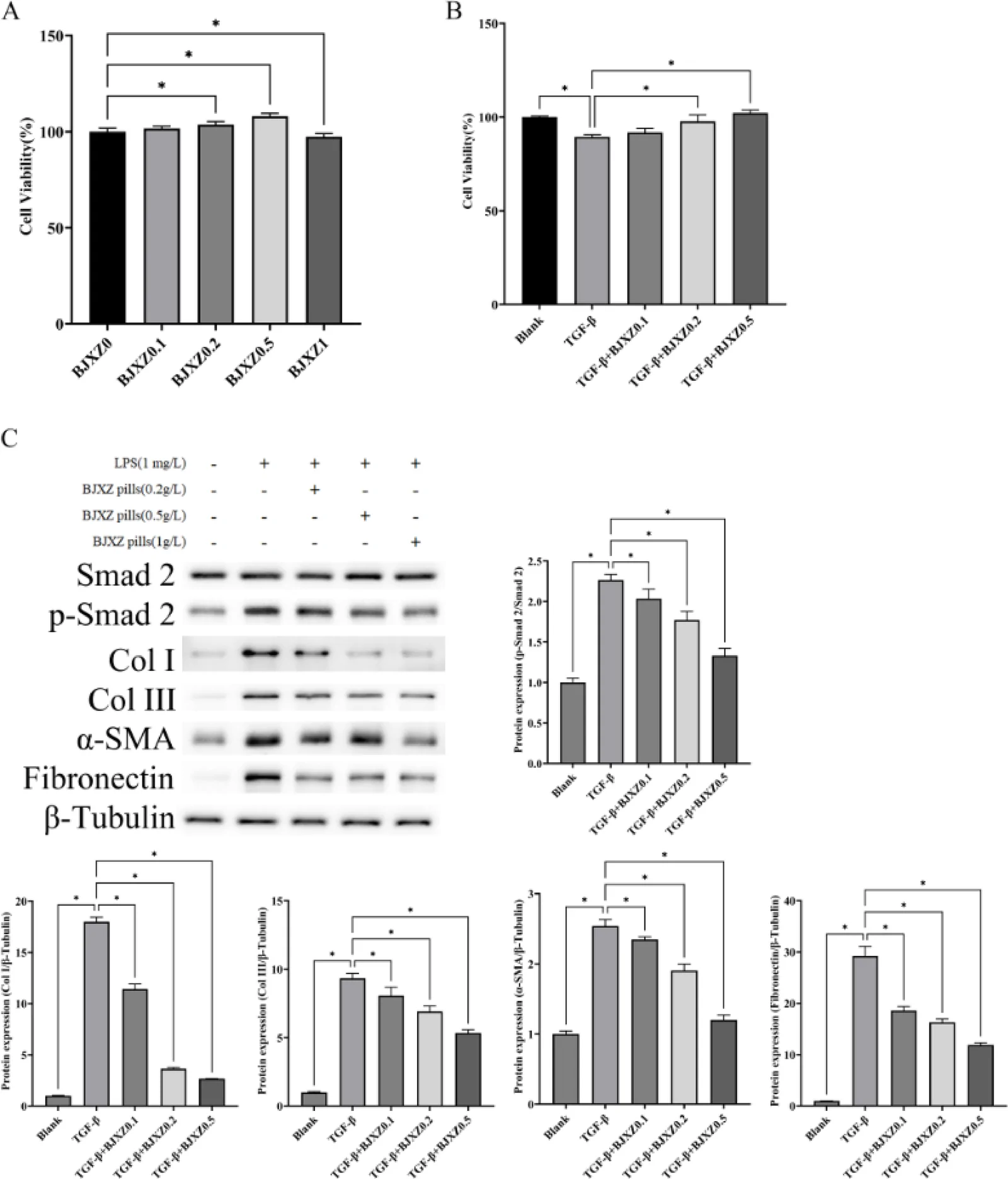
Effect of Biejiaxiaozheng pills on TGF-β-induced activation model of LX-2 cells. (**A**) Effect of Biejiaxiaozheng pills on the viability of LX-2 cells. (**B**) Effect of Biejiaxiaozheng pills on the Viability of TGF-β-induced activation model of LX-2 cells. (**C**) Effect of Biejiaxiaozheng pills on the fibrosis-related proteins Expression of LX-2 cells.

After 48 h of treatment, BJXZ pills at concentrations of 0.2 and 0.5 g·L^−1^ significantly enhanced the viability of LX-2 cells. However, increasing the dosage to 1 g·L^-1^ resulted in a marked reduction in cell viability. Therefore, subsequent efficacy studies were conducted at a concentration of 0.5 g·L^−1^ (Fig. 5A). Following 48 h of TGF-β induction, LX-2 cells exhibited significantly reduced viability. Treatment with BJXZ pills at concentrations of 0.2 and 0.5 g·L^−1^ markedly restored LX-2 cell viability, suggesting that BJXZ pills may exert protective effects against TGF-β-induced suppression of cell viability (Fig. 5B). Quantitative analysis revealed that TGF-β stimulation for 48 h significantly upregulated the phosphorylation levels of Smad 2 (p-Smad 2) in LX-2 cells. Additionally, there was a substantial increase in the expression of extracellular matrix components, including collagen type I, collagen type III, α-SMA, and fibronectin. Notably, BJXZ treatment demonstrated a concentration-dependent inhibitory effect on TGF-β-induced fibrotic protein expression, with maximal suppression observed at the concentration 0.5 g·L^−1^ BJXZ pills (Fig. 5C).

## Discussion

Liver cirrhosis caused by viral hepatitis and alcoholic liver disease causes 1 million deaths every year globally ^15,16^. Liver fibrosis is a liver scarring response caused by hepatocyte damage by one or more external factors and is an intermediate stage in the progression of chronic liver disease to cirrhosis and liver cancer. The occurrence and progression of liver fibrosis is associated with chemokines ^17,18^, inflammation ^19,20^, oxidative stress ^21,22^, and neuroendocrine pathways ^23,24^. After liver injury has occurred, apoptotic hepatocyte DNA induces the activation of hepatic stellate cells and activates liver repair ^25,26^. Following that, intrahepatic macrophages are activated and amphiregulin (AREG) ^27^ or cadherin-11 ^28^ mediates activation of upregulated TGF-β1 to induce differentiation of fibroblasts towards myofibroblasts and Kupffer cells towards fibrocytes ^29,30^. This continuously promotes collagen synthesis and deposition and ultimately leads to liver fibrosis. Studies have shown that inflammation-related pathways can be effectively targeted for the diagnosis and treatment of liver fibrosis ^31^, and many traditional Chinese medicines have been proven to be effective clinical treatments of renal fibrosis ^32^, liver fibrosis ^33^, pulmonary fibrosis ^34^, and myocardial fibrosis ^35^ by inhibiting inflammation.

Biejiaxiaozheng (BJXZ) pills is a compound preparation composed of 11 traditional Chinese medicines developed by our team. BJXZ pills are used to treat portal hypertension and liver fibrosis and have been used as an adjuvant therapy for hepatitis B. BJXZ pills have been used in clinical practice for more than 30 years and exhibit significant efficacy on liver diseases. Our team previously constructed a rat liver fibrosis model by using carbon tetrachloride and demonstrated that BJXZ pills have anti-liver fibrosis effects, and the underlying mechanism may be associated with anti-inflammatory and antioxidative stress effects ^7^. In order to further study the mechanisms of action of BJXZ pills in liver fibrosis treatment, we employed network pharmacology in this study to analyze the anti-liver fibrosis effects of BJXZ pills. The results of the constructed PPI network showed that the anti-liver fibrosis effects may be associated with IL1B, IL6, TP53, CASP3, STAT1, JUN, and FOS, and KEGG enrichment analysis showed that these effects may be related to pathways upstream and downstream of TNFα. Network pharmacology analysis results and previous animal experiment results ^7,8^ both showed that the anti-liver fibrosis effects of BJXZ pills are associated with inhibition of inflammation. Therefore, our team further constructed an in vitro inflammatory model using LPS-induced RAW 264.7 macrophages and an in vitro hepatic stellate cell (HSC) activation model using TGF-β-induced LX-2 cells. These models were utilized to simulate the inflammatory activation of Kupffer cells and the activation of hepatic stellate cells in liver fibrosis, respectively. We aimed to validate the inhibitory effects of BJXZ pills on the activation of Kupffer cells and hepatic stellate cells, and to further explore the underlying mechanisms.

The results from the in vitro experiments showed that BJXZ pills inhibit the increased cell viability of activated RAW 264.7 cells after stimulation with 1 mg·L^−1^ LPS, and inhibit the phosphorylation of P65 and the expression of INOS, thus reducing the content of NO, TNF-α, IL-1β, IL-6 and MCP-1 induced by LPS in the culture medium. BJXZ pills also effectively controlled the inflammatory response and blocked the inflammatory cascade to prevent a cytokine storm. Studies have shown a rapid release of a considerable number of inflammatory mediators, such as TNF-α, IL-1β, and IL-6, during acute inflammation, which causes tissue oxygen consumption, respiratory burst, and synthesis of a large amount of ROS ^36^. This can effectively eliminate foreign objects within a short period of time. Hence, ROS is an important downstream signal of TNF-α ^37,38^. However, excessive and uncontrolled ROS is an important factor of inflammation-induced tissue injury, and abnormally elevated ROS levels can further activate the NF-κB pathway to carry out proinflammatory effects ^39^. Nrf2 serves as a master regulator of the endogenous antioxidant defense system, orchestrating multiple cytoprotective signaling pathways involved in cellular defense mechanisms. HO-1, a downstream effector of Nrf2, exerts cytoprotective effects through its ability to mitigate oxidative damage, regulate apoptotic processes, and modulate inflammatory responses. Under conditions of elevated intracellular oxidative stress, excessive ROS induce the dissociation of Nrf2 from its cytoplasmic inhibitor Keap1. The liberated Nrf2 translocates into the nucleus, where it transcriptionally upregulates HO-1 expression, thereby enhancing the cell’s antioxidant capacity against oxidative stress ^40^. Previous studies have demonstrated that upregulating Nrf2 expression while simultaneously inhibiting INOS expression alleviates hepatic inflammation, representing an effective therapeutic mechanism for reversing hepatic fibrosis ^41^. DCFH-DA assay found that intracellular ROS significantly increased in RAW 264.7 cells after LPS induction, and BJXZ pills could significantly decrease the intracellular ROS levels in a dose-dependent manner. The mechanism underlying this antioxidant effect may be due to increases in Nrf2 and HO-1 expression. Our study findings indicate that BJXZ pills can inhibit the phosphorylation of P65 and the expression of INOS, increase the expression of Nrf2 and HO-1, thereby counteracting the activation of the NF-κB pathway and the synthesis of ROS induced by LPS, ultimately alleviating the inflammatory response and the tissue damage it causes. However, the underlying mechanism of BJXZ pills in upregulating Nrf2 expression requires further exploration.

During inflammatory activation of macrophages, ROS can act as a downstream signal of TNFα and can also originate from macrophage M1 polarization to cause the Warburg effect ^42–44^. Due to the Warburg effect, ATP synthesis is decreased, resulting in a decrease in the intracellular ATP/ADP ratio, which may ultimately lead to macrophage apoptosis and further aggravate the inflammatory response. Our results showed that after LPS-induced (1 mg·L^−1^ LPS) M1 polarization of RAW 264.7 cells, the mitochondrial membrane potential (MMP) and ATP content within the cells were significantly reduced. BJXZ pills intervention restored the MMP and ATP content in a dose-dependent manner, and the mechanism may be related to the fact that BJXZ pills can inhibit DRP1 and restore the expression of OPA1, indicating that BJXZ pills can improve the energy metabolism of M1 polarized macrophages.

Hepatic stellate cells (HSCs) are key effector cells responsible for the excessive deposition of extracellular matrix (ECM) during hepatic fibrogenesis. In their quiescent state, HSCs primarily function as vitamin A-storing cells; however, upon stimulation by inflammatory cytokines such as TGF-β and TNF-α, they undergo activation and transdifferentiate into myofibroblast-like cells. Activated HSCs exhibit enhanced proliferative, contractile, pro-inflammatory, and chemotactic properties, contributing to ECM remodeling through the synthesis of α-SMA, collagen, and other matrix components, thereby participating in wound healing and fibrotic scar formation ^45^.

Mitochondrial damage-associated molecular patterns (mito-DAMPs) released from hepatocyte injury and TGF-β or platelet-derived growth factor (PDGF) secreted by activated macrophages can directly activate HSCs ^46,47^. Our previous studies ^7^ have demonstrated that BJXZ pills protect against TGF-β-induced hepatocyte injury, while the present study further confirms that BJXZ pills significantly suppress LPS-induced macrophage activation. Therefore, we employed a TGF-β-induced LX-2 cell activation model to investigate whether BJXZ pills exert anti-fibrotic effects by directly inhibiting hepatic stellate cell activation. The results showed that BJXZ pills significantly reduced Smad 2 phosphorylation, suppressed TGF-β/Smad signaling pathway activation, and thereby decreased the expression of fibrotic markers including α-SMA, Collagen I, Collagen III, and fibronectin, ultimately achieving anti-fibrotic effects.

## Conclusions

Network pharmacology identified multiple mechanisms underlying Biejiaxiaozheng pills’ anti-liver fibrosis effects, with the TNF signaling pathway as a key contributor. To validate these findings, we established an LPS-induced RAW 264.7 macrophage inflammation model and a TGF-β-induced LX-2 hepatic stellate cell (HSC) activation model. Results showed that the pills significantly inhibited NF-κB signaling, reducing inflammatory cytokine expression, while enhancing NRF2 pathway activity to alleviate oxidative stress. Additionally, they regulated mitochondrial function, improved energy metabolism, suppressed HSC activation, and downregulated fibrosis markers. Mechanistically, the pills alleviated liver fibrosis by inhibiting both Kupffer cells (macrophages) and HSCs. These findings demonstrate that Biejiaxiaozheng Pills exert anti-fibrotic effects via multi-target regulation of inflammation, oxidative stress, and HSC activation, supporting their therapeutic potential for liver fibrosis.

## Electronic supplementary material

Below is the link to the electronic supplementary material.

Supplementary Material 1

Supplementary Material 2

## Data Availability

The original contributions presented in the study are included in the article. Further inquiries can be directed to the corresponding authors.
