# Supplementary material for: A network pharmacology approach confirms Biejiaxiaozheng pills combat hepatic fibrosis by modulating macrophage inflammation and hepatic stellate cell activation
Source: Sci Rep. 2025 Jul 9;15:24638. doi: 10.1038/s41598-025-09002-1 (PMC12241332; doi:10.1038/s41598-025-09002-1)
Supplement: Supplementary file 1 — Supplementary Material 1 [file 41598_2025_9002_MOESM1_ESM.docx]

Supplementary Table 1. The active ingredient of the herb medicines contained in Biejiaxiaozheng Pills

| **Herb** | **Mol ID** | **Molecule Name** | **OB (%)** | **DL** |
| --- | --- | --- | --- | --- |
| *Atractylodes macrocephala* Koidz. | MOL000020 | 12-senecioyl-2E,8E,10E-atractylentriol | 62.4 | 0.22 |
|  | MOL000021 | 14-acetyl-12-senecioyl-2E,8E,10E-atractylentriol | 60.31 | 0.31 |
|  | MOL000022 | 14-acetyl-12-senecioyl-2E,8Z,10E-atractylentriol | 63.37 | 0.3 |
|  | MOL000028 | α-Amyrin | 39.51 | 0.76 |
|  | MOL000033 | (3S,8S,9S,10R,13R,14S,17R)-10,13-dimethyl-17-[(2R,5S)-5-propan-2-yloctan-2-yl]-2,3,4,7,8,9,11,12,14,15,16,17-dodecahydro-1H-cyclopenta[a]phenanthren-3-ol | 36.23 | 0.78 |
|  | MOL000049 | 3β-acetoxyatractylone | 54.07 | 0.22 |
|  | MOL000072 | 8β-ethoxy atractylenolide Ⅲ | 35.95 | 0.21 |
| *Poria cocos* (Schw.) Wolf | MOL000273 | (2R)-2-[(3S,5R,10S,13R,14R,16R,17R)-3,16-dihydroxy-4,4,10,13,14-pentamethyl-2,3,5,6,12,15,16,17-octahydro-1H-cyclopenta[a]phenanthren-17-yl]-6-methylhept-5-enoic acid | 30.93 | 0.81 |
|  | MOL000275 | trametenolic acid | 38.71 | 0.8 |
|  | MOL000276 | 7,9(11)-dehydropachymic acid | 35.11 | 0.81 |
|  | MOL000279 | Cerevisterol | 37.96 | 0.77 |
|  | MOL000280 | (2R)-2-[(3S,5R,10S,13R,14R,16R,17R)-3,16-dihydroxy-4,4,10,13,14-pentamethyl-2,3,5,6,12,15,16,17-octahydro-1H-cyclopenta[a]phenanthren-17-yl]-5-isopropyl-hex-5-enoic acid | 31.07 | 0.82 |
|  | MOL000282 | ergosta-7,22E-dien-3beta-ol | 43.51 | 0.72 |
|  | MOL000283 | Ergosterol peroxide | 40.36 | 0.81 |
|  | MOL000285 | (2R)-2-[(5R,10S,13R,14R,16R,17R)-16-hydroxy-3-keto-4,4,10,13,14-pentamethyl-1,2,5,6,12,15,16,17-octahydrocyclopenta[a]phenanthren-17-yl]-5-isopropyl-hex-5-enoic acid | 38.26 | 0.82 |
|  | MOL000287 | 3beta-Hydroxy-24-methylene-8-lanostene-21-oic acid | 38.7 | 0.81 |
|  | MOL000289 | pachymic acid | 33.63 | 0.81 |
|  | MOL000290 | Poricoic acid A | 30.61 | 0.76 |
|  | MOL000291 | Poricoic acid B | 30.52 | 0.75 |
|  | MOL000292 | poricoic acid C | 38.15 | 0.75 |
|  | MOL000296 | hederagenin | 36.91 | 0.75 |
|  | MOL000300 | dehydroeburicoic acid | 44.17 | 0.83 |
| *Rheum palmatum* L. | MOL002235 | EUPATIN | 50.8 | 0.41 |
|  | MOL002251 | Mutatochrome | 48.64 | 0.61 |
|  | MOL002259 | Physciondiglucoside | 41.65 | 0.63 |
|  | MOL002260 | Procyanidin B-5,3'-O-gallate | 31.99 | 0.32 |
|  | MOL002268 | rhein | 47.07 | 0.28 |
|  | MOL002276 | Sennoside E_qt | 50.69 | 0.61 |
|  | MOL002280 | Torachrysone-8-O-beta-D-(6'-oxayl)-glucoside | 43.02 | 0.74 |
|  | MOL002281 | Toralactone | 46.46 | 0.24 |
|  | MOL002288 | Emodin-1-O-beta-D-glucopyranoside | 44.81 | 0.8 |
|  | MOL002293 | Sennoside D_qt | 61.06 | 0.61 |
|  | MOL002297 | Daucosterol_qt | 35.89 | 0.7 |
|  | MOL002303 | palmidin A | 32.45 | 0.65 |
|  | MOL000358 | beta-sitosterol | 36.91 | 0.75 |
|  | OL000471 | aloe-emodin | 83.38 | 0.24 |
|  | MOL000554 | gallic acid-3-O-(6'-O-galloyl)-glucoside | 30.25 | 0.67 |
|  | MOL000096 | (-)-catechin | 49.68 | 0.24 |
| *Astragalus membranaceus*(Fisch.) Bge.var. *mongholicus*(Bge.) Hsiao | MOL000211 | Mairin | 55.38 | 0.78 |
|  | MOL000239 | Jaranol | 50.83 | 0.29 |
|  | MOL000296 | hederagenin | 36.91 | 0.75 |
|  | MOL000033 | (3S,8S,9S,10R,13R,14S,17R)-10,13-dimethyl-17-[(2R,5S)-5-propan-2-yloctan-2-yl]-2,3,4,7,8,9,11,12,14,15,16,17-dodecahydro-1H-cyclopenta[a]phenanthren-3-ol | 36.23 | 0.78 |
|  | MOL000354 | isorhamnetin | 49.6 | 0.31 |
|  | MOL000371 | 3,9-di-O-methylnissolin | 53.74 | 0.48 |
|  | MOL000374 | 5'-hydroxyiso-muronulatol-2',5'-di-O-glucoside | 41.72 | 0.69 |
|  | MOL000378 | 7-O-methylisomucronulatol | 74.69 | 0.3 |
|  | MOL000379 | 9,10-dimethoxypterocarpan-3-O-β-D-glucoside | 36.74 | 0.92 |
|  | MOL000380 | (6aR,11aR)-9,10-dimethoxy-6a,11a-dihydro-6H-benzofurano[3,2-c]chromen-3-ol | 64.26 | 0.42 |
|  | MOL000387 | Bifendate | 31.1 | 0.67 |
|  | MOL000392 | formononetin | 69.67 | 0.21 |
|  | MOL000398 | isoflavanone | 109.99 | 0.3 |
|  | MOL000417 | Calycosin | 47.75 | 0.24 |
|  | MOL000422 | kaempferol | 41.88 | 0.24 |
|  | MOL000433 | FA | 68.96 | 0.71 |
|  | MOL000438 | (3R)-3-(2-hydroxy-3,4-dimethoxyphenyl)chroman-7-ol | 67.67 | 0.26 |
|  | MOL000439 | isomucronulatol-7,2'-di-O-glucosiole | 49.28 | 0.62 |
|  | MOL000442 | 1,7-Dihydroxy-3,9-dimethoxy pterocarpene | 39.05 | 0.48 |
|  | MOL000098 | quercetin | 46.43 | 0.28 |
| *Glycyrrhiza uralensis* Fisch. | MOL001484 | Inermine | 75.18 | 0.54 |
|  | MOL001792 | DFV | 32.76 | 0.18 |
|  | MOL000211 | Mairin | 55.38 | 0.78 |
|  | MOL002311 | Glycyrol | 90.78 | 0.67 |
|  | MOL000239 | Jaranol | 50.83 | 0.29 |
|  | MOL002565 | Medicarpin | 49.22 | 0.34 |
|  | MOL002844 | Pinocembrin | 64.72 | 0.18 |
|  | MOL000354 | isorhamnetin | 49.6 | 0.31 |
|  | MOL000359 | sitosterol | 36.91 | 0.75 |
|  | MOL003656 | Lupiwighteone | 51.64 | 0.37 |
|  | MOL003896 | 7-Methoxy-2-methyl isoflavone | 42.56 | 0.2 |
|  | MOL000392 | formononetin | 69.67 | 0.21 |
|  | MOL000417 | Calycosin | 47.75 | 0.24 |
|  | MOL000422 | kaempferol | 41.88 | 0.24 |
|  | MOL004328 | naringenin | 59.29 | 0.21 |
|  | MOL004805 | (2S)-2-[4-hydroxy-3-(3-methylbut-2-enyl)phenyl]-8,8-dimethyl-2,3-dihydropyrano[2,3-f]chromen-4-one | 31.79 | 0.72 |
|  | MOL004806 | euchrenone | 30.29 | 0.57 |
|  | MOL004808 | glyasperin B | 65.22 | 0.44 |
|  | MOL004810 | glyasperin F | 75.84 | 0.54 |
|  | MOL004811 | Glyasperin C | 45.56 | 0.4 |
|  | MOL004814 | Isotrifoliol | 31.94 | 0.42 |
|  | MOL004815 | (E)-1-(2,4-dihydroxyphenyl)-3-(2,2-dimethylchromen-6-yl)prop-2-en-1-one | 39.62 | 0.35 |
|  | MOL004820 | kanzonols W | 50.48 | 0.52 |
|  | MOL004824 | (2S)-6-(2,4-dihydroxyphenyl)-2-(2-hydroxypropan-2-yl)-4-methoxy-2,3-dihydrofuro[3,2-g]chromen-7-one | 60.25 | 0.63 |
|  | MOL004827 | Semilicoisoflavone B | 48.78 | 0.55 |
|  | MOL004828 | Glepidotin A | 44.72 | 0.35 |
|  | MOL004829 | Glepidotin B | 64.46 | 0.34 |
|  | MOL004833 | Phaseolinisoflavan | 32.01 | 0.45 |
|  | MOL004835 | Glypallichalcone | 61.6 | 0.19 |
|  | MOL004838 | 8-(6-hydroxy-2-benzofuranyl)-2,2-dimethyl-5-chromenol | 58.44 | 0.38 |
|  | MOL004841 | Licochalcone B | 76.76 | 0.19 |
|  | MOL004848 | licochalcone G | 49.25 | 0.32 |
|  | MOL004849 | 3-(2,4-dihydroxyphenyl)-8-(1,1-dimethylprop-2-enyl)-7-hydroxy-5-methoxy-coumarin | 59.62 | 0.43 |
|  | MOL004855 | Licoricone | 63.58 | 0.47 |
|  | MOL004856 | Gancaonin A | 51.08 | 0.4 |
|  | MOL004857 | Gancaonin B | 48.79 | 0.45 |
|  | MOL004860 | licorice glycoside E | 32.89 | 0.27 |
|  | MOL004863 | 3-(3,4-dihydroxyphenyl)-5,7-dihydroxy-8-(3-methylbut-2-enyl)chromone | 66.37 | 0.41 |
|  | MOL004864 | 5,7-dihydroxy-3-(4-methoxyphenyl)-8-(3-methylbut-2-enyl)chromone | 30.49 | 0.41 |
|  | MOL004866 | 2-(3,4-dihydroxyphenyl)-5,7-dihydroxy-6-(3-methylbut-2-enyl)chromone | 44.15 | 0.41 |
|  | MOL004879 | Glycyrin | 52.61 | 0.47 |
|  | MOL004882 | Licocoumarone | 33.21 | 0.36 |
|  | MOL004883 | Licoisoflavone | 41.61 | 0.42 |
|  | MOL004884 | Licoisoflavone B | 38.93 | 0.55 |
|  | MOL004885 | licoisoflavanone | 52.47 | 0.54 |
|  | MOL004891 | shinpterocarpin | 80.3 | 0.73 |
|  | MOL004898 | (E)-3-[3,4-dihydroxy-5-(3-methylbut-2-enyl)phenyl]-1-(2,4-dihydroxyphenyl)prop-2-en-1-one | 46.27 | 0.31 |
|  | MOL004903 | liquiritin | 65.69 | 0.74 |
|  | MOL004904 | licopyranocoumarin | 80.36 | 0.65 |
|  | MOL004905 | 3,22-Dihydroxy-11-oxo-delta(12)-oleanene-27-alpha-methoxycarbonyl-29-oic acid | 34.32 | 0.55 |
|  | MOL004907 | Glyzaglabrin | 61.07 | 0.35 |
|  | MOL004908 | Glabridin | 53.25 | 0.47 |
|  | MOL004910 | Glabranin | 52.9 | 0.31 |
|  | MOL004911 | Glabrene | 46.27 | 0.44 |
|  | MOL004912 | Glabrone | 52.51 | 0.5 |
|  | MOL004913 | 1,3-dihydroxy-9-methoxy-6-benzofurano[3,2-c]chromenone | 48.14 | 0.43 |
|  | MOL004914 | 1,3-dihydroxy-8,9-dimethoxy-6-benzofurano[3,2-c]chromenone | 62.9 | 0.53 |
|  | MOL004915 | Eurycarpin A | 43.28 | 0.37 |
|  | MOL004917 | glycyroside | 37.25 | 0.79 |
|  | MOL004924 | (-)-Medicocarpin | 40.99 | 0.95 |
|  | MOL004935 | Sigmoidin-B | 34.88 | 0.41 |
|  | MOL004941 | (2R)-7-hydroxy-2-(4-hydroxyphenyl)chroman-4-one | 71.12 | 0.18 |
|  | MOL004945 | (2S)-7-hydroxy-2-(4-hydroxyphenyl)-8-(3-methylbut-2-enyl)chroman-4-one | 36.57 | 0.32 |
|  | MOL004948 | Isoglycyrol | 44.7 | 0.84 |
|  | MOL004949 | Isolicoflavonol | 45.17 | 0.42 |
|  | MOL004957 | HMO | 38.37 | 0.21 |
|  | MOL004959 | 1-Methoxyphaseollidin | 69.98 | 0.64 |
|  | MOL004961 | Quercetin der. | 46.45 | 0.33 |
|  | MOL004966 | 3'-Hydroxy-4'-O-Methylglabridin | 43.71 | 0.57 |
|  | MOL000497 | licochalcone a | 40.79 | 0.29 |
|  | MOL004974 | 3'-Methoxyglabridin | 46.16 | 0.57 |
|  | MOL004978 | 2-[(3R)-8,8-dimethyl-3,4-dihydro-2H-pyrano[6,5-f]chromen-3-yl]-5-methoxyphenol | 36.21 | 0.52 |
|  | MOL004980 | Inflacoumarin A | 39.71 | 0.33 |
|  | MOL004985 | icos-5-enoic acid | 30.7 | 0.2 |
|  | MOL004988 | Kanzonol F | 32.47 | 0.89 |
|  | MOL004989 | 6-prenylated eriodictyol | 39.22 | 0.41 |
|  | MOL004990 | 7,2',4'-trihydroxy－5-methoxy-3－arylcoumarin | 83.71 | 0.27 |
|  | MOL004991 | 7-Acetoxy-2-methylisoflavone | 38.92 | 0.26 |
|  | MOL004993 | 8-prenylated eriodictyol | 53.79 | 0.4 |
|  | MOL004996 | gadelaidic acid | 30.7 | 0.2 |
|  | MOL000500 | Vestitol | 74.66 | 0.21 |
|  | MOL005000 | Gancaonin G | 60.44 | 0.39 |
|  | MOL005001 | Gancaonin H | 50.1 | 0.78 |
|  | MOL005003 | Licoagrocarpin | 58.81 | 0.58 |
|  | MOL005007 | Glyasperins M | 72.67 | 0.59 |
|  | MOL005008 | Glycyrrhiza flavonol A | 41.28 | 0.6 |
|  | MOL005012 | Licoagroisoflavone | 57.28 | 0.49 |
|  | MOL005013 | 18α-hydroxyglycyrrhetic acid | 41.16 | 0.71 |
|  | MOL005016 | Odoratin | 49.95 | 0.3 |
|  | MOL005017 | Phaseol | 78.77 | 0.58 |
|  | MOL005018 | Xambioona | 54.85 | 0.87 |
|  | MOL005020 | dehydroglyasperins C | 53.82 | 0.37 |
|  | MOL000098 | quercetin | 46.43 | 0.28 |
| *Panax quiquefolium* L. | MOL011394 | (2R,3S,4S,5R,6R)-2-(hydroxymethyl)-6-[[(3S,5R,8R,9R,10R,12R,13R,14R,17S)-12-hydroxy-4,4,8,10,14-pentamethyl-17-[(2S)-6-methyl-2-[(2S,3R,4S,5S,6R)-3,4,5-trihydroxy-6-(hydroxymethyl)oxan-2-yl]oxyhept-5-en-2-yl]-2,3,5,6,7,9,11,12,13,15,16,17-dodecahydro-1H-c | 36.43 | 0.25 |
|  | MOL011434 | polyacetylene PQ-2 | 36.74 | 0.2 |
|  | MOL011435 | PQ-2 | 36.74 | 0.19 |
|  | MOL011442 | (8S,9S,10R,13R,14S,17R)-17-[(1R,4R)-4-ethyl-1,5-dimethylhexyl]-10,13-dimethyl-1,2,8,9,11,12,14,15,16,17-decahydrocyclopenta[a]phenanthren-7-one | 43.87 | 0.75 |
|  | MOL011455 | 20-Hexadecanoylingenol | 32.7 | 0.65 |
|  | MOL000358 | beta-sitosterol | 36.91 | 0.75 |
|  | MOL005344 | ginsenoside rh2 | 36.32 | 0.56 |
|  | MOL006774 | stigmast-7-enol | 37.42 | 0.75 |
|  | MOL006980 | papaverine | 64.04 | 0.38 |
|  | MOL008173 | daucosterol_qt | 36.91 | 0.75 |
|  | MOL008397 | Daturilin | 50.37 | 0.77 |
| *Curcua kwangsiensis* S.G.Lee et C.Fliang | MOL000296 | hederagenin | 36.91 | 0.75 |
|  | MOL000906 | wenjine | 47.93 | 0.27 |
|  | MOL000940 | bisdemethoxycurcumin | 77.38 | 0.26 |
| *Sparganium stoloniferum* Buch.-Ham | MOL001297 | trans-gondoic acid | 30.7 | 0.2 |
|  | MOL000296 | hederagenin | 36.91 | 0.75 |
|  | MOL000358 | beta-sitosterol | 36.91 | 0.75 |
|  | MOL000392 | formononetin | 69.67 | 0.21 |
|  | MOL000449 | Stigmasterol | 43.83 | 0.76 |
| *Paeonia lactiflora* Pall. | MOL001910 | 11alpha,12alpha-epoxy-3beta-23-dihydroxy-30-norolean-20-en-28,12beta-olide | 64.77 | 0.38 |
|  | MOL001918 | paeoniflorgenone | 87.59 | 0.37 |
|  | MOL001919 | (3S,5R,8R,9R,10S,14S)-3,17-dihydroxy-4,4,8,10,14-pentamethyl-2,3,5,6,7,9-hexahydro-1H-cyclopenta[a]phenanthrene-15,16-dione | 43.56 | 0.53 |
|  | MOL001921 | Lactiflorin | 49.12 | 0.8 |
|  | MOL001924 | paeoniflorin | 53.87 | 0.79 |
|  | MOL001925 | paeoniflorin_qt | 68.18 | 0.4 |
|  | MOL001928 | albiflorin_qt | 66.64 | 0.33 |
|  | MOL001930 | benzoyl paeoniflorin | 31.27 | 0.75 |
|  | MOL000211 | Mairin | 55.38 | 0.78 |
|  | MOL000358 | beta-sitosterol | 36.91 | 0.75 |
|  | MOL000359 | sitosterol | 36.91 | 0.75 |
|  | MOL000422 | kaempferol | 41.88 | 0.24 |
|  | MOL000492 | (+)-catechin | 54.83 | 0.24 |

Supplementary Table 2. The active ingredient of the animal medicines contained in Biejiaxiaozheng Pills

| **animal medicine** | **Ingredient id** | **Ingredient name** |
| --- | --- | --- |
| **musk** | HBIN001991 | 17-beta-estradiol |
|  | HBIN002351 | 1β,2β,5α,11-tetraacetoxy-8α-benzoyl-4α-hydrox-y-7β-nicotinoyl-dihydroagarofuran |
|  | HBIN003690 | 22-cyclopentyloxil-22-deisopentyl-3beta-hydroxyl-guranstanol |
|  | HBIN004870 | 2,6-decamethylene pyridine |
|  | HBIN004958 | 2,6-Nonamethylene pyridine |
|  | HBIN007627 | 3,5-dihydroxybenzoicacid |
|  | HBIN007902 | 3alpha,17-dihydroxy-5beta-androstane |
|  | HBIN007947 | 3alpha-hydroxy-5alpha-androstan-17-one |
|  | HBIN007948 | 3alpha-hydroxy-5beta-androstan-17-one |
|  | HBIN007952 | 3alpha-hydroxy-androst-4-ene-17-one |
|  | HBIN007969 | 3α-ureido-androst-4-en-17β-ol |
|  | HBIN007970 | 3α-ureido-androst-4-en-17-one |
|  | HBIN008037 | 3beta,17alpha-dihydroxy-5alpha-androstane |
|  | HBIN008231 | 3beta-hydroxy-5alpha-androstan-17-one |
|  | HBIN008237 | 3beta-hydroxy-androst-5-ene-17-one |
|  | HBIN020391 | cholest-4-ene-3-one |
|  | HBIN020400 | cholesterol |
|  | HBIN020402 | Cholesteryl ferulate |
|  | HBIN022377 | cyclotetradecan-1-one |
|  | HBIN022389 | cyclovirobuxine |
|  | HBIN022898 | decamine |
|  | HBIN025818 | estragole |
|  | HBIN029742 | hydroxymuscopyridine a |
|  | HBIN029743 | hydroxymuscopyridine b |
|  | HBIN035746 | morin |
|  | HBIN008930 | 3-methylcyclotridecan-1-one |
|  | HBIN009720 | 3'(s)-hydroxy-4'(r)-angeloyloxy-3',4'-dihydro-xanthyletin |
|  | HBIN011406 | 5 alpha-androstan-3,17-dione |
|  | HBIN011407 | 5α-androstane-3β,17α-diol |
|  | HBIN011458 | 5 beta-androstan-3,17-dione |
|  | HBIN011459 | 5 beta-androstan-3 alpha,17 alpha-diol |
|  | HBIN011460 | 5 beta-androstan-3 alpha,17 beta-diol |
|  | HBIN011503 | 5-cis-cyclopentadecen-1-one |
|  | HBIN011504 | 5-cis-cyclotetradecen-1-one |
|  | HBIN012459 | 6-Hydroxy-musizin-8-O-beta-D-glucoside |
|  | HBIN015193 | allantoin |
|  | HBIN015508 | alpha estradiol |
|  | HBIN016029 | androst-4,6-diene-3,17-dione |
|  | HBIN016031 | androst-4-ene-3,17-dione |
|  | HBIN016036 | androsterone |
|  | HBIN018098 | beta-estradiol |
|  | HBIN036015 | musclide a1 |
|  | HBIN036016 | muscol |
|  | HBIN036017 | muscone |
|  | HBIN036018 | muscopyridine |
|  | HBIN036019 | musennin |
|  | HBIN037160 | n-nonane |
|  | HBIN037169 | n-nornuciferine |
|  | HBIN037365 | normuscone |
|  | HBIN044201 | s-methyl cysteine |
|  | HBIN046010 | testosterone |
| ***Trionyx sinensis* Wiegmann** | HBIN000481 | 11-Octadecenoic acid |
|  | HBIN013193 | 7-Hexadecanoic acid |
|  | HBIN013530 | 8,11-OCTADECADIENOIC ACID |
|  | HBIN013853 | 8-Octadecenoic acid |
|  | HBIN013995 | 9,12-Octadecadienoic acid |
|  | HBIN014102 | 9-Hexadecanoic acid |
|  | HBIN014155 | 9-Octadecenoic acid |
|  | HBIN000418 | 11-eicosenoicacid |
|  | HBIN021284 | coloagen |
|  | HBIN029081 | heptadecanoic acid |
|  | HBIN029268 | hexadecanoic acid |
|  | HBIN032132 | keratin |
|  | HBIN037749 | octadecanoic acid |
|  | HBIN039143 | pentadecanoic acid |
|  | HBIN046037 | tetradecanoic acid |
|  | HBIN048048 | vitamin d |
