# Supplementary material for: A network pharmacology approach confirms Biejiaxiaozheng pills combat hepatic fibrosis by modulating macrophage inflammation and hepatic stellate cell activation
Source: Sci Rep. 2025 Jul 9;15:24638. doi: 10.1038/s41598-025-09002-1 (PMC12241332; doi:10.1038/s41598-025-09002-1)
Supplement: Supplementary file 2 — Supplementary Material 2 [file 41598_2025_9002_MOESM2_ESM.pdf]

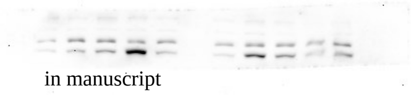

in manuscript

FIG 2C Original data of Western blotting images of INOS

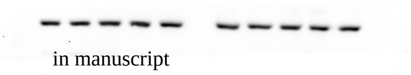

in manuscript

FIG 2C Original data of Western blotting images of β-Tubulin

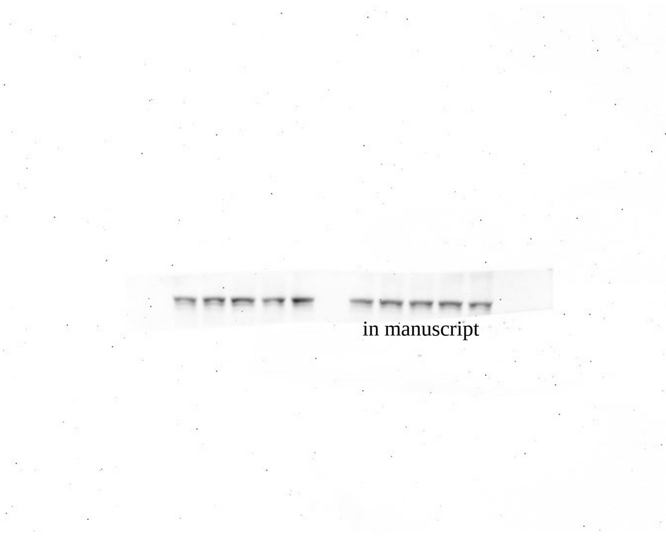

FIG 2C Original data of Western blotting images of P65

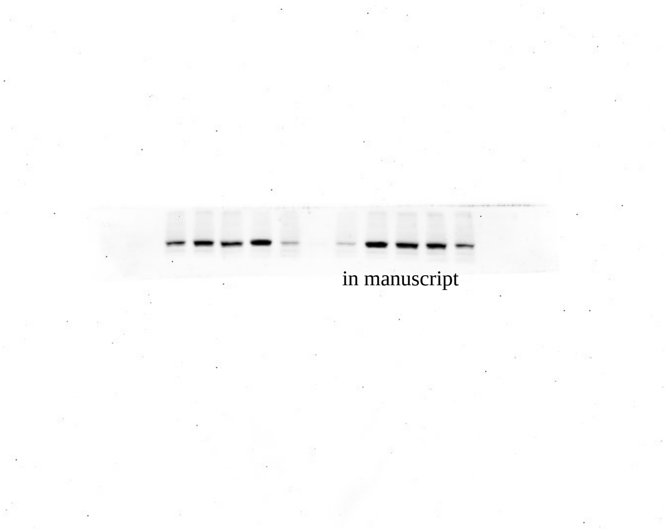

FIG 2C Original data of Western blotting images of p-P65

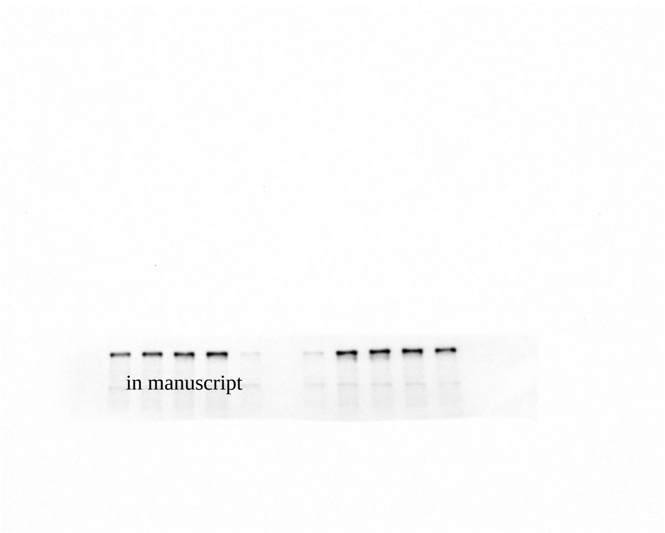

FIG 2C Original data of Western blotting images of p-P65

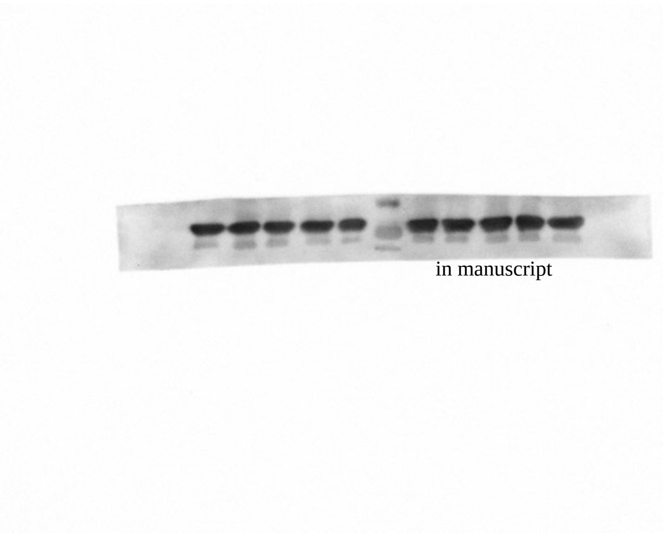

FIG 2C Original data of Western blotting images of Histone H3

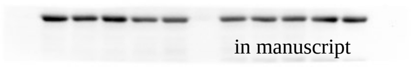

FIG 3B Original data of Western blotting images of Nrf2

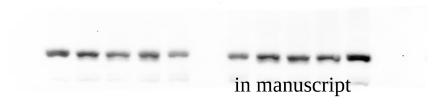

FIG 3B Original data of Western blotting images of HO-1

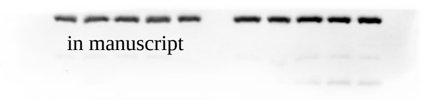

FIG 3B Original data of Western blotting images of β-Tubulin

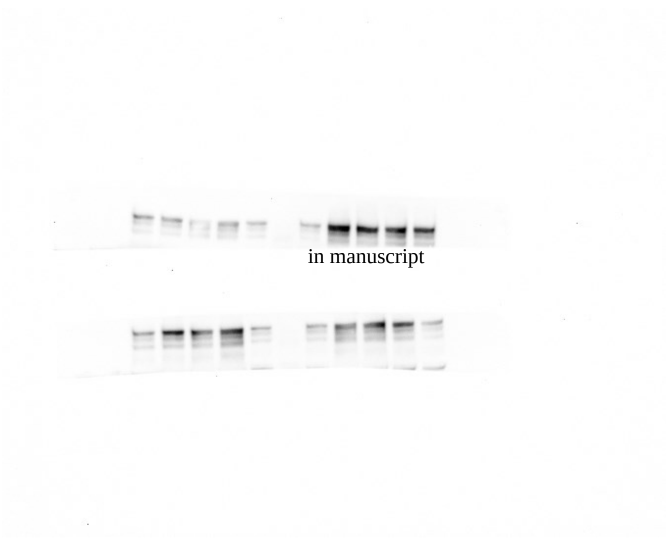

FIG 4C Original data of Western blotting images of DRP1

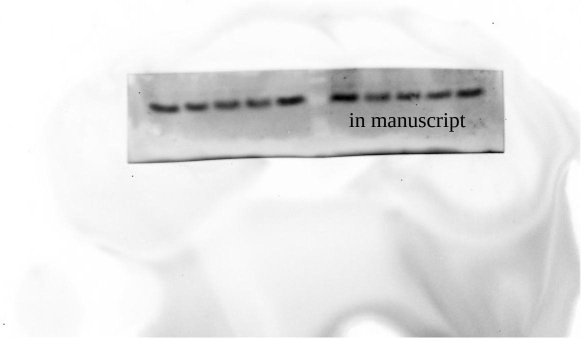

FIG 4C Original data of Western blotting images of OPA

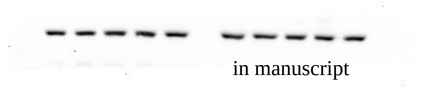

FIG 4C Original data of Western blotting images of β-Tubulin

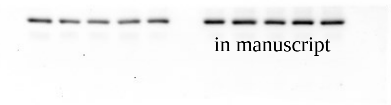

FIG 5C Original data of Western blotting images of Smad 2

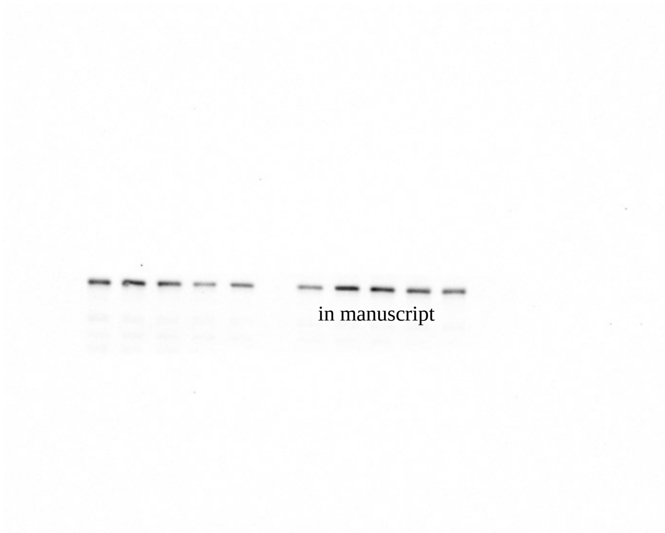

FIG 5C Original data of Western blotting images of p-Smad 2

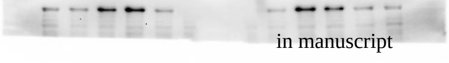

FIG 5C Original data of Western blotting images of Col I

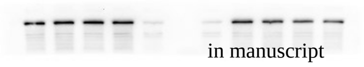

FIG 5C Original data of Western blotting images of Col III

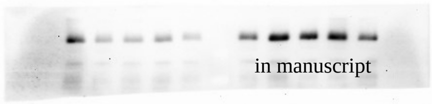

FIG 5C Original data of Western blotting images of α-SMA

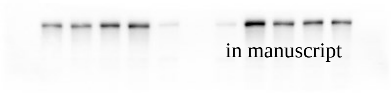

FIG 5C Original data of Western blotting images of Fibronectin

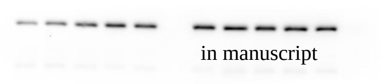

FIG 5C Original data of Western blotting images of β-Tubulin
